# Gender differences in Reasons for Sickness Presenteeism - a study among GPs in a Swedish health care organization

**DOI:** 10.1186/s40557-016-0136-x

**Published:** 2016-09-20

**Authors:** Marie Gustafsson Sendén, Karin Schenck-Gustafsson, Ann Fridner

**Affiliations:** 1 Department of psychology, Stockholm University, 106 91 Stockholm, Sweden; 2 Karolinska Institute, Stockholm, Sweden

**Keywords:** Sickness presenteeism, Gender, Gender stereotypes, Health at work

## Abstract

**Background:**

It is common that physicians go to work while sick and therefore it is important to understand the reasons behind. Previous research has shown that women and men differ in health and health related behavior. In this study, we examine gender differences among general practitioners who work while sick.

**Methods:**

General practitioners (GP’s) working in outpatient care in a Swedish city participated in the study (*n* = 283; women = 63 %; response rate = 41 %). Data were obtained from a large web-based questionnaire about health and organization within primary care. Two questions about sickness presenteeism (going to work while sick) were included; life-long and during the past 12 months, and five questions about reasons. We controlled for general health, work-family conflict and demographic variables.

**Results:**

Female physicians reported sickness presenteeism more often than male physicians. Work-family conflict mediated the association between gender and sickness presenteeism.

Women reported reasons related with “concern for others” and “workload” more strongly than men. Men reported reasons related with “capacity” and “money” more strongly than women. These differences are likely effects of gender stereotyping and different family-responsibilities.

**Conclusions:**

Gender socialization and gender stereotypes may influence work and health-related behavior. Because sickness presenteeism is related with negative effects both on individuals and at organizational levels, it is important that managers of health organizations understand the reasons for this, and how gender roles may influence the prevalence of sickness presenteeism and the reasons that female and male GPs give for their behavior.

## Background

Sickness presenteeism is defined as going to work while sick, and is common in the health sector and among physicians [1–3]. Studies typically report a prevalence of 80 %–90 % among physicians [4–10], which can be compared to 30 %–70 % in other professions [11, 12].

It is shown that working through illness is associated with future long-term sick leave as well as coronary heart disease [13, 14]. For the health-care system, sickness presenteeism leads to costs in terms of medical errors, productivity loss and reduced empathy with patients [15–18]. In order to prevent sickness presenteeism, it is important to understand the reasons why physicians work when they are sick, and also whether there are any gender differences in the reasons given.

Two studies from Great Britain [3] and New Zeeland [2] explored reasons for presenteeism among physicians. Among British GPS, concern about increased workload of colleagues was the most common reason, followed by concern for booked patients, and work piling up. In qualitative interviews, physicians expressed that “giving in to illness may also be perceived as weakness” [3]. New Zealand GPs reported that taking sick leave was unfair to colleagues. Other reasons were difficulties of replacement, work piling up, not being sick enough, and pressure from the workplace [2]. What remains to be studied, is whether there are gender differences in the reasons given.

Empirical studies on gender differences in sickness presenteeism show conflicting results; some find higher presenteeism among women than men [1, 11, 19, 20], while others find no gender differences [5, 9]. It has also been suggested that the family situation influences women’s health more than men’s, because of different family burden and its effect [21]. Being married has been related with health problems in women, but to fewer health problems in men [22]. Sick leave among women has been related with women’s greater family responsibilities, and research from Sweden shows that illness among women increases after the second child is born [23]. Sweden is considered as one of the most gender equal countries in the world [24]. Yet, studies show higher number of sickness absenteeism and presenteeism among women [1, 19, 25]. It has been argued that gender equality in the work place, but not in the family could explain the paradox that gender equality in Sweden has not paid off in terms of better health among women [23].

### Gender differences in reasons given for sickness presenteeism

The reasons given for sickness presenteeism may differ because of socialization and gender stereotypes. Women are socialized to be relationship-oriented and focus on others more than themselves. Boys and men are socialized to be independent, competitive, and the breadwinner [26, 27]. Hence, both the male and the female gender stereotypes could encourage sickness presenteeism, but the mechanisms may differ. The male stereotype could influence reasons related with ability, money and not giving in to weakness. For example, male physicians report less help-seeking behavior than female physicians because of gender stereotypes [28, 29]. The female stereotype, on the other hand, may results in reasons emphasizing relations with others (such as colleagues and patients). That ‘work piles up’ was a common reason given by physicians in previous research [2, 3]. If women experience higher work family conflict, they might also report reasons about work piling up more strongly than men.

Two aspects of sickness presenteeism and gender will be analyzed in the current study: prevalence and reasons reported. The hypotheses are:

- H1: Female GPs report higher rates of sickness presenteeism than men.
- H2: Work-family conflict can explain the relation between gender differences in sickness presenteeism.
- H3a: Reasons related with others and work load are expressed more strongly by female than male GPs.
- H3b: Reasons related with strength and money are expressed more strongly by male than female GPs.

## Method

The present study used baseline cross-sectional data from a primary care organization in Sweden. All general practitioners who were permanently employed and actively working (*n* = 698; women = 61 %) were invited to participate in the study. From the total sample, 283 general practitioners (women = 63 %) participated by completing a web-based questionnaire (response rate = 41 %). The age of the respondents was categorized in three groups: younger than 44 (40.6 %), 45–54 (27.2 %) and older than 55 (32.5 %). The age or gender distribution did not differ from the total population. The sample included residents (28.2 %), specialists (64.0 %) and chief physicians (7.8 %). Chief physicians were somewhat overrepresented in this sample (5 % in population).

The data collection was completed through a web survey including approximately 100 questions related with health and work environment. Information about the project was distributed to employees by researchers, the CEO of the organization and the chair of the employee organization. All participants received an email with a URL-address and a password to log in to the survey. Five reminders were sent out after the first email. The regional ethics board approved the study^1^.

### Variables

*Two measures of presenteeism were used:*

1. *Life-long sickness presenteeism* was measured by the question: “Have you ever gone to work with an illness for which you would have recommended a patient to stay at home?” Reponses were given on a 5-point scale (1 = very seldom or never, 5 = very often or always).
2. *Sickness presenteeism during the last 12-month* period was assessed through the question: “How often has this happened during the last 12-month period?” Response alternatives were (0 = never, 1 = once, 2 = 2–4 times, 3 = more than five times). These measures have been used before in research on sickness presenteeism [1, 8, 12].

*Work-family conflict* was measured with one question from the QPS Nordic [30]:”Do the demands of your work interfere with your home and family life?” ?” Reponses were given on a 5-point scale (1 = very seldom or never, 5 = very often or always).

*Reasons for sickness presenteeism* were measured by five items. These responses were derived from previous research [2, 3] and a pilot study within the current organization. Physicians who indicated that they had gone to work were given a follow-up question: “What were the reasons for you going to work while you were sick?” with five response options: 1) “I do not want to burden my colleagues”, 2) “I do not want to burden the patients”, 3) “The work is piling up and I will need to do it when I go back”, 4) “I feel that I can handle it”, 5) “I lose money if I stay at home”.

*General health* was included as a control variable, and was assessed by the question: “How is your general health (physical and mental) in comparison to other people of your age? Responses were given on a 5-point scale (1 = very good, 5 = very bad).

Demographic variables such as gender, marital status, number of children and position were also included as control variables.

### Analyses

Gender differences in presenteeism were analyzed through univariate ANOVAs. Mediation analyses were computed by the process command developed by Hayes [31]. Analyzes of reasons were computed with a MANOVA. In this analysis, we excluded 39 physicians who indicated they never went to work when sick.

We controlled for demographic factors such as marital status, number of children and age, self-rated health and work-family conflict. Because, there was a slight overrepresentation of chief physicians among respondents, we also computed the analyses with chief physicians excluded.

Throughout this article, *p*-values of .05 or less are considered significant. All analyses were performed with SPSS, version 22.

## Results

Descriptive statistics of the sample are included in Table 1.

**Table 1:** Descriptive statistics of the sample included in the study (*n* = 283)

	Women		Men		All	*p-*value
	%		%		%	
Age						.260
<=44	44.2		34.3		40.5	
45-54	26		29.4		27.2	
> = 55	29.8		36.3		32.2	
Position						.400
Residents	30.9		23.5		28.2	
Specialist	61.9		57.5		64.0	
Chief physicians	7.2		8.8		7.8	
Marital status						.483
Married/or cohabiting	82.3		84.3		83.0	
In a relation, not cohabiting^a^	3.9		5.9		4.6	
Single^*^	13.8		9.8		12.4	
	*M*	*SD*	*M*	*SD*	*M*	
Number kids	1.97	1.05	1.83	1.22	1.92	.313
Working Time	39.85	10.43	41.27	8.12	40.36	.238
Work-family conflict	3.10	1.08	2.75	1.06	2.97	.008
General Health	3.76	0.87	3.63	0.97	3.71	.256

*Gender differences were tested by chi-square analyses on frequencies and *t*-test for means

^a^In Sweden it is common that romantic partners do not live together. Therefor, this marital status was included in the questionnaire

**Fig. 1:**
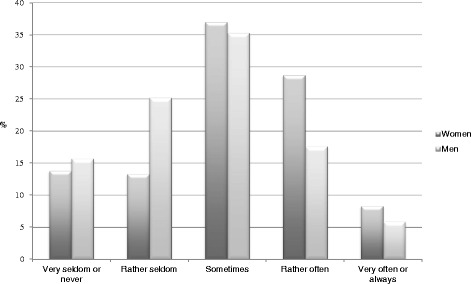
Responses given to the question “Have you ever gone to work with an illness for which you would have recommended a patient to stay at home?” (*n* = 283)

A univariate ANOVA, with gender as the independent variable, sickness presenteeism as the dependent variable, and self-reported general health as a covariate, showed a significant difference between women and men, F (1, 289) = 75.95, p = .006, *ŋ*_p_^2^ = .026, such that women (M = 3.07, SD = 1.12) reported sickness presenteeism more often than men (M = 2.73, SD = 1.11). Figure 1 shows for example that 74 % of the women and 59 % of the men stated that they “sometimes too often” go to work when they are sick.

**Fig. 2:**
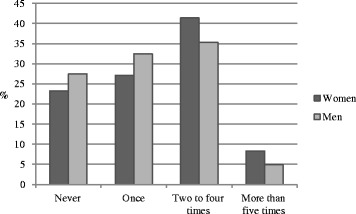
Responses given to the question How often have this happened during the last 12 month period?” (*n* = 283)

We also computed a univariate ANOVA for sickness presenteeism during the last 12-month period, with gender as the independent variable and general health as covariate. There was a marginally significant effect of gender, *F* (1, 280) = 2.711, *p* = 0.065, *ŋ*_p_^2^ = .012; women (*M* = 1.35; *SD* = 0.93) reported presenteeism more often than men (*M* = 1.18, *SD* = 0.86). Figure 2, shows that 49 % of the women and 40 % of the men indicated they had worked while sick more than twice over a 12-month period.

**Fig. 3:**
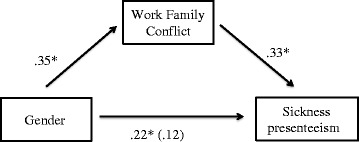
Standardized regression coefficients for the relationship between gender and sickness presenteeism as mediated by work-family conflict. The standardized regression coefficient between gender and sickness presenteeism, controlling for work-family conflict, is in parentheses * *p* <05

The process macro for mediation analyses [31] was used to investigate the hypothesis that work-family conflict mediates gender differences in life-long sickness presenteeism. First, in support of a meditational hypothesis, it was shown that gender was a significant predictor of work-family conflict, *b* = .35, *SE* = .13, *p* < .05, and that work-family conflict was a significant predictor of life-long presenteeism, *b* = .33, *SE* = .06, *p* < .05 (see Fig. 3). Second, and consistent with full mediation, when both the mediator and gender was inserted in the model, gender was no longer a significant predictor of sickness presenteeism, *b* = .22, *SE* = .13, *ns*. Approximately 12 % of the variance in satisfaction was accounted for by the predictors (*R*^*2*^ = .12). The indirect effect was tested using a bootstrap estimation approach with 1000 samples. The bootstrapped unstandardized indirect effect was .22, and the 95 % confidence interval ranged from .04 to .23. Thus the indirect effect was statistically significant.

A MANOVA, including the five reasons for going to work while sick as the dependent variables, gender as the independent variable, and general health as a covariate, showed a main effect of gender, F(5, 236) = 5.076, *p* < 0.01, *ŋ*^2^ = 0.097. Table 2 includes the means, standard deviations and results from the post hoc univariate analyses. All arguments revealed gender differences in expected direction. The arguments related with “money loss” and “strength” were significantly stronger arguments for men than women, whereas “concern for patients” and “work piles up” were significantly stronger arguments for women than men. Women also showed somewhat more “concern for colleagues”, but this difference was only marginally significant (*p* = .084).^2^

**Table 2:** Reasons for sickness presenteeism as indicated by women and men

	Women (*n* = 158)		Men (*n* = 85)			
	*M*	*SD*	*M*	*SD*	*p*	ŋ _*p*_^2^
Work piles up	4.30	0.95	3.91	1.09	0.004	0.039
Concern for colleagues	3.76	0.99	3.52	1.11	0.082	0.012
Concern for patients	3.67	1.07	3.32	1.16	0.018	0.023
Can handle it	3.11	0.89	3.39	0.89	0.019	0.029
Money loss	2.18	1.20	2.64	1.25	0.006	0.030

*P*-values and effect measures indicate gender differences as measured through univariate analyses (*n* = 243)

## Discussion

This study aimed to examine how gender is related with sickness presenteeism and reasons behind. In support of hypotheses, we found that women more often go to work when sick than men, and that women and men also differ in the reasons they give for this behavior. We found that women have higher prevalence of sickness presenteeism, both in a long- and a short-term perspective. Previous research has shown that gender differences in illness-related behavior are due to different professions, different status and differences in the family situation [22, 32, 33]. In our study, there were no differences in marital status, number of children or position. All participants also had the same employer. Neither did the overall gender distribution of men and women within the profession offer any explanation, because women were in majority (women = 61 %). However, work-family conflict offered an explanation. Work-family conflict was more strongly expressed by women than men, and it also mediated the differences between gender and sickness presenteeism. Thus, experiencing work-family conflict could explain women’s higher prevalence.

Our hypothesis about ‘female’ reasons for sickness presenteeism was partially supported. Women showed a greater concern for patients and colleagues than men. However, the difference in concern for colleagues was only marginally significant (*p* = .084). That “work piles up” was the most important reason given by both women and men, though to a higher degree among women. All these reasons might be associated with the female stereotype in which women learn to focus on others more than on them selves.

The hypothesis about ‘male reasons was fully supported in the sense that men expressed reasons associated with the male sterotype (i.e. ability and money) more strongly than women. This result is convergent with how male physicians avoid expressions of weakness [28, 29]. The reasons given are also related with the male stereotype of being a breadwinner [34, 35].

The reasons associated with a stereotypical female gender role were altogether more strongly held by both women and men than reasons associated with the stereotypical male gender role. These reasons were also most common in previous research among GPs [2, 3]. Future research could study whether arguments differ among physician specialties, for example if physicians in stereotypically male specialties report more stereotypically male reasons.

### Implications for managers of health-care organizations

Sickness presenteeism is negative both for health care organizations and physicians themselves [13, 14, 36]. We suggest that managers of organizations inform physicians about the short- and long-term consequences of sickness presenteeism. For example, guidelines about health behavior could be included in organizational policies or work contracts. It could clearly be stated that physicians should stay at home while sick.

It is important for health-care managers to emphasize that concerns about colleagues are valuable within the organization, but that is shall not lead to working while sick. Previous research has shown that working when sick instead increases the burden of colleagues, and leads to a deterioration in relations with patients as well as with colleagues [15, 17]. For contagious diseases, the negative consequences are obvious.

Reasons concerning money are more difficult to resolve within an organization. However, policies could inform that a short-term loss of money can, in fact, lead to long-term gains in both money and health. Because also work-family conflict is associated with sickness presenteeism, it also needs also to be discussed within an organization.

### Limitations

The present study relied on self-reported measures, which may lead to problems associated with inflating the strength of relationships and to common methodological response biases. We computed a Harman’s one factor test [37], which showed that common-source variance was not a problem in this study.

The response rate was 41 %, which is quite low, but rather common in organizational research among highly educated and busy professionals [38]. Still a relative high number of doctors were involved (*n* = 283). A comparison of age and gender distribution among participants showed no systematic differences in responses and population.

Another limitation is that we did not assess gender roles at the individual level, but rather deduced that biological sex is associated with gender roles. In a study on gender differences in morbidity [22], the Bem Social Role Inventory [39] was used. This study found that gender roles explained a higher variance in self-reported health than biological gender. This possibility could be investigated in future research, by including both sex of respondent and gender role identity.

Measuring sickness presenteeism is based on self-reports because there are no objective tests to use. When physicians report how often this has happened, it is important to note that these reports include errors related with memories and differences in how sickness presenteeism is defined. A strength in studies on sickness presenteeism among physicians, is that physicians are asked to relate their own health to how they would advice patients in the same situation.

The strength of this study is that it compares women and men in an organization that is equal in terms of positions, age, sex distribution. Much research on gender differences has found differences that might be related with women and men having jobs of different status.

## Conclusion

This study is the first to examine gender differences in sickness presenteeism and reasons behind. We found that female physicians report sickness presenteeism more often than male physicians, and that gender socialization and roles could be related with the reasons given. Because physicians show great concern for their colleagues and patients, we advise managers of medical organizations to encourage physicians to care for themselves.

## Abbreviations

GP: General practitioner

## Footnotes

1 Ethical approval registration number: 2012/1500-32 (EPN Stockholm)

2 Because previous research has shown that marital status and number of children have different effects for men and women we also included marital status and number of children as interaction variables in all analyses of variance. None of these interactions were significant. We also included “work-family-conflict” as a covariate, and this factor did not change any of the gender differences in reasons given.
